# Supplementary material for: A Large-Scale Multiple Genome Comparison of Acidophilic Archaea (pH ≤ 5.0) Extends Our Understanding of Oxidative Stress Responses in Polyextreme Environments
Source: Antioxidants (Basel). 2021 Dec 28;11(1):59. doi: 10.3390/antiox11010059 (PMC8773360; doi:10.3390/antiox11010059)
Supplement: Supplementary file 1 [file antioxidants-11-00059-s001.zip › supplementary_figures.pdf]

```
                                                                     Tyr41
  Saccharolobus solfataricus 98/2    MT-LQIQFKKYELPPLPYKIDALEPYISKDIIDVHYNGHHKGYVNGANSLLERLEKVVKG
Sulfolobus acidocaldarius DSM 639    MT-QVIQLKRYEFPQLPYKVDALEPYISKDIIDVHYNGHHKGYVNGANSLLDRLEKLIKG
     Acidianus brierleyi DSM 1651    MS-SSISFKKYELPPLPYKLDALEPYISKDIIDLHYNGHHKGYVNGANSFLDRMQKVTKG
   Metallosphaera sedula DSM 5348    MS-SSISFKKFELPPLPYKVDALEPYISKDIIDVHYNGHHKGYVTGANTFMERFNKVIKG
            Thermoproteus sp. AZ2    MA---VQFKKYELPPLPYNLDALEPYISRDIVDVHYNGHHKGYVNTANSLLDRLEKIIRG
 Caldivirga maquilingensis IC-167    MA-AQTLFKRYELPPLPYNVNALEPYISGQVIDVHYNGHHKGYVNGANAAIDRLEKIIKN
Vulcanisaeta distributa DSM 14429    MSLPATLFKRYELPPLPYSINALEPHISGQVIDVHYNGHHKGYVNGANATIERLEKIIKG
Thermocladium modestius JCM 10088    MS-SANLFKRYELPPLPYKTSDLEPHISAQVIDVHYNGHHKGYVNGANATVDRLEKILKG
 Acidilobus saccharovorans 345-15    MV----SLKRYELPPLPYNYDALEPIISAETLRYHHDKHHLGYVNGANAALDKLEKYLNG
Caldisphaera lagunensis DSM 15908    MV----SYKRYELPPLPYSYDALEPVLSRDILTYHHDKHHLAYVNGANAAMEKLEKYLNG
                                     *       *::*:* ***. . *** :* : :  *:: ** .**. **: :::::*  ..

  Saccharolobus solfataricus 98/2    DLQTGQYDIQGIIRGLTFNINGHKLHALYWENMAPSGKGGGKPGGALADLINKQYGSFDR
Sulfolobus acidocaldarius DSM 639    DLPQGQYDLQGILRGLTFNINGHKLHAIYWNNMAPAGKGGGKPGGALADLINKQYGSFDR
     Acidianus brierleyi DSM 1651    ELSSGQYDIQGLLRGLVFNINGHKLHSLYWENMAPNGKGGGKPGGSLADLIEKQYGSFDK
   Metallosphaera sedula DSM 5348    ELQSGQYDVQGLMRGIVFNINGHKLHALYWDNMAPAGKGGGQPGGALADLIVKQYGSYDR
            Thermoproteus sp. AZ2    ELGQGQYDIQGILRGLTFNINGHKLHDLYWKSMAPASKGGGKPGGRLADLINKQYGSFDR
 Caldivirga maquilingensis IC-167    EVTS--YDIQGLLRNLFFNINGHKLHTLYWNSMAPAGKGGGTPGGYLGDLVIKQFGSYDK
Vulcanisaeta distributa DSM 14429    DVTS--YDIQGLLRSLFFNVNGHKLHTLYWYSMAPPGKGGGTPGGYLGDLIKKQFGSFDR
Thermocladium modestius JCM 10088    DVTS--YDIQGLLRNLFFNINGHKLHAIYWHSMAPSGKGGGKPGGKLADLITKQYGGYDK
 Acidilobus saccharovorans 345-15    QLTD--IDVRAVSRDFEFNYGGHILHTLYWLNMAPKGKGGGTPGGAIGDAINKFFGSFDK
Caldisphaera lagunensis DSM 15908    QEQS--IDIRAVSRDFEFNYGGHLLHTLYWLNMAPTGKGGGTPGGTIADAINKNFGSFDK
                                     :      *::.: *.: ** .** ** :** .*** .**** *** :.* : * :*.:*:
                                                                His155
  Saccharolobus solfataricus 98/2    FKQVFTETANSLPGTGWAVLYYDTESGNLQIMTFENHFQNHIAEIPIILILDEFEHAYYL
Sulfolobus acidocaldarius DSM 639    FKQVFSESANSLPGSGWTVLYYDNESGNLQIMTVENHFMNHIAELPVILIVDEFEHAYYL
     Acidianus brierleyi DSM 1651    FKALFTEAANSLPGTGWTVLYYETENGNLQIMTFENHFQNHIAELPIVLILDEFEHAYYL
   Metallosphaera sedula DSM 5348    FKQVFTETANSLPGTGWTVLYYDTENGNLEIMTFENHFMNHIAELPILLILDEFEHAYYL
            Thermoproteus sp. AZ2    FKAVFTEAANSLPGTGWTALYYDTETGNLQIMTFENHFLNHIGEAPILLIVDEFEHAYYL
 Caldivirga maquilingensis IC-167    FRNLFNEVMRSLPGSGWAVLYYDTETGNLVFTTFENHYNQHIAELPVILIIDEFEHAYYL
Vulcanisaeta distributa DSM 14429    FKALFTEVMRSLPGCGWTVLYYDPETGNLEFTTFENHYNQHIAELPILLIVDEFEHAYYL
Thermocladium modestius JCM 10088    FRKIFDEVMRSLPGSGWATLYYDPETGNLVFTTFENHYNQHIAELPVLLIVDEFEHAYYL
 Acidilobus saccharovorans 345-15    FKKLFGDAAKNVEGVGWAILAYDPVTGDLRILQVEKHNNVVTTNLIPLLAVDVFEHAYYI
Caldisphaera lagunensis DSM 15908    FKKVFGDAAKLVEGVGWAILALDPVTGDLKITQVEKHNAVITMNLVPLLACDVFEHAYYL
                                     *: :* :  . : * **: *  :  .*:* :  .*:*      :   :*  * ******:

  Saccharolobus solfataricus 98/2    QYKNKRADYVNAWWNVVNWDAAEKKLQKYLTK-----
Sulfolobus acidocaldarius DSM 639    QYKNKRGDYLNAWWNVVNWDDAEKRLQKYLNK-----
     Acidianus brierleyi DSM 1651    QYKNKRADYVNAWWNLVNWDYANKKLEKYLNK-----
   Metallosphaera sedula DSM 5348    QYKNKRADYVTNWWNVVNWDYAEKKLQKYLK------
            Thermoproteus sp. AZ2    QYKNKRADYVNAWWNLVNWDYAEQKLSKLL-------
 Caldivirga maquilingensis IC-167    QYKNNRNAYLDAIWNVLNWGEAENRLRKYIK------
Vulcanisaeta distributa DSM 14429    QYRSNRNGYIDAIWNVLNWEEAENRLRKYIK------
Thermocladium modestius JCM 10088    QYKNNRNAYLDAIWNVLNWEEAEKRLSKYL-------
 Acidilobus saccharovorans 345-15    DYRNDRAKYVDSWWDLINWDDVEARYQKALNTPKLIL
Caldisphaera lagunensis DSM 15908    QYKNDRGSYVDKWWDVVNWDDVEKRYQKALTLPKLIL
                                     :*:..*  *:   *:::**  .: :  * :
```

Figure S1. Multiple sequence alignment of Crenarchaeota Fe-SOD. In red are emphasized the conserved key amino acids Tyr41 and His155 through the different sequences.

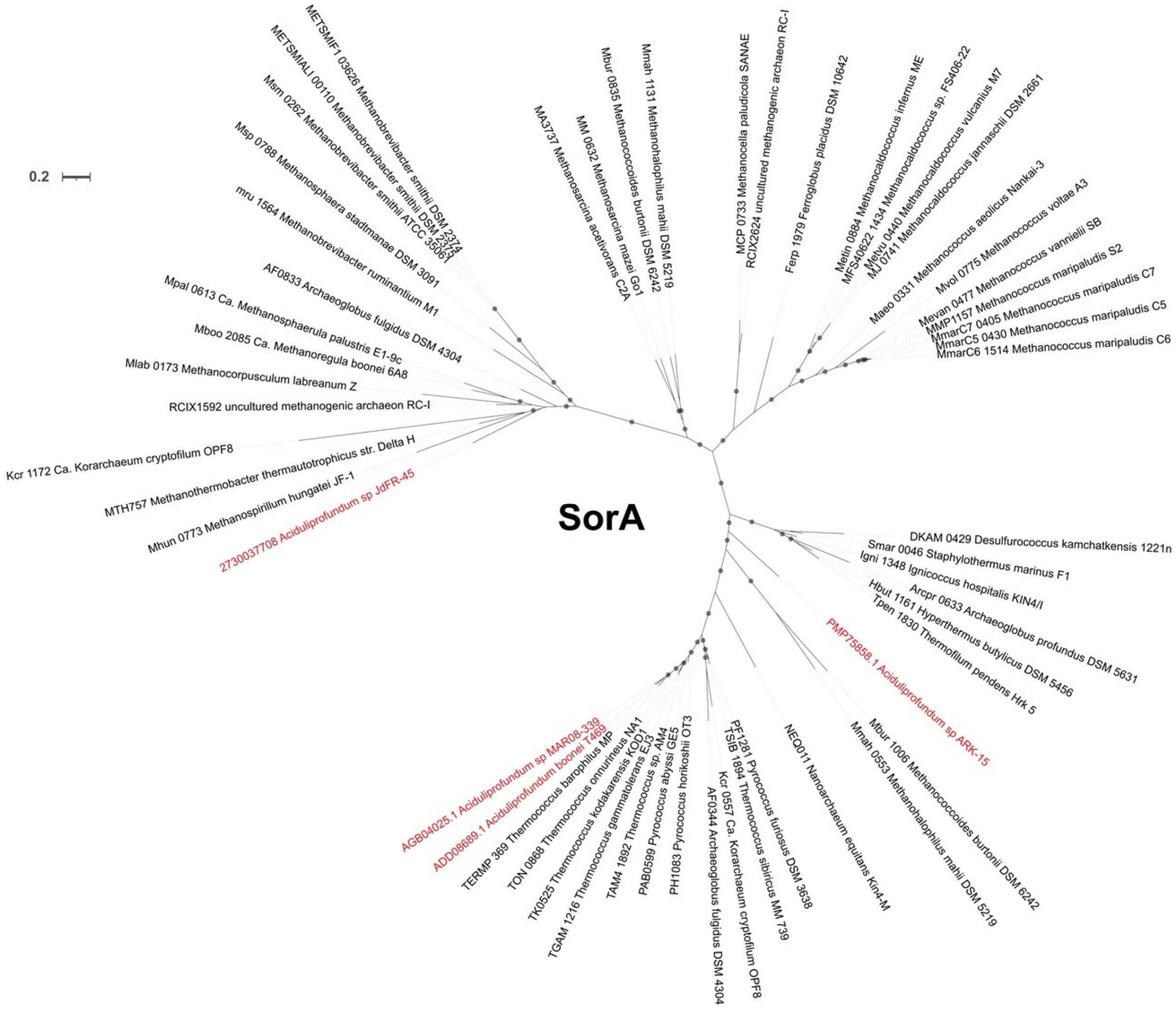

Figure S2. Unrooted phylogenetic tree constructed from the predicted amino acid sequences of SorA in *Aciduliprofundum* and Archaeal sequences from sorGOdb. In red color are sequences from *Aciduliprofundum.* Bootstrap values over 60 are represented with gray circles in the respective branches. Scale bar represent 0.2 amino acid substitutions per site.

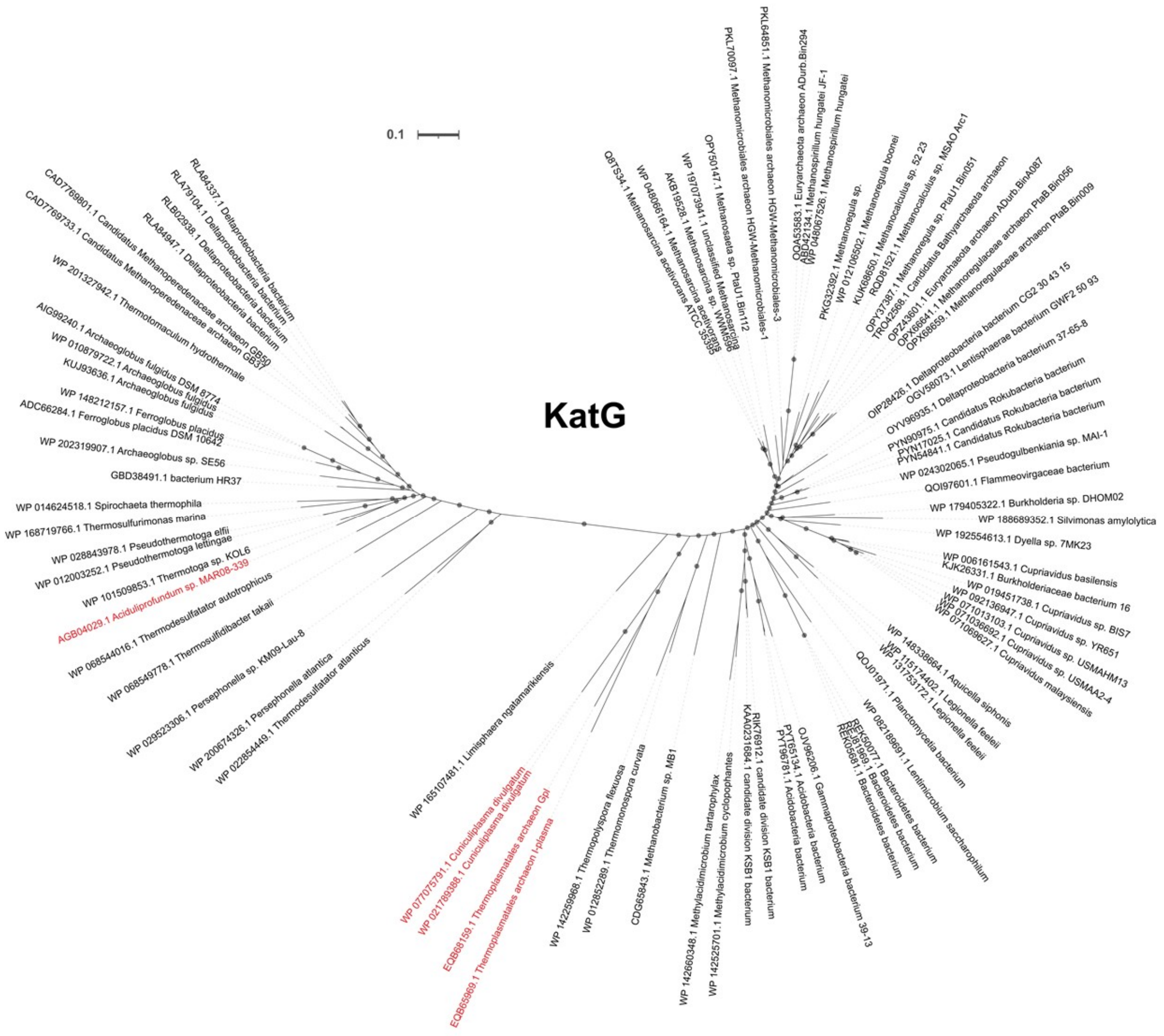

Figure S3: Unrooted phylogenetic tree constructed from the predicted amino acid sequences of KatG in *Aciduliprofundum, Cuniculiplasma* and *Thermoplasmatales* and their best hits in the NCBI. In red color are sequences from *Aciduliprofundum.* Bootstrap values over 60 are represented with gray circles in the respective branches. Scale bar represent 0.1 amino acid substitutions per site.

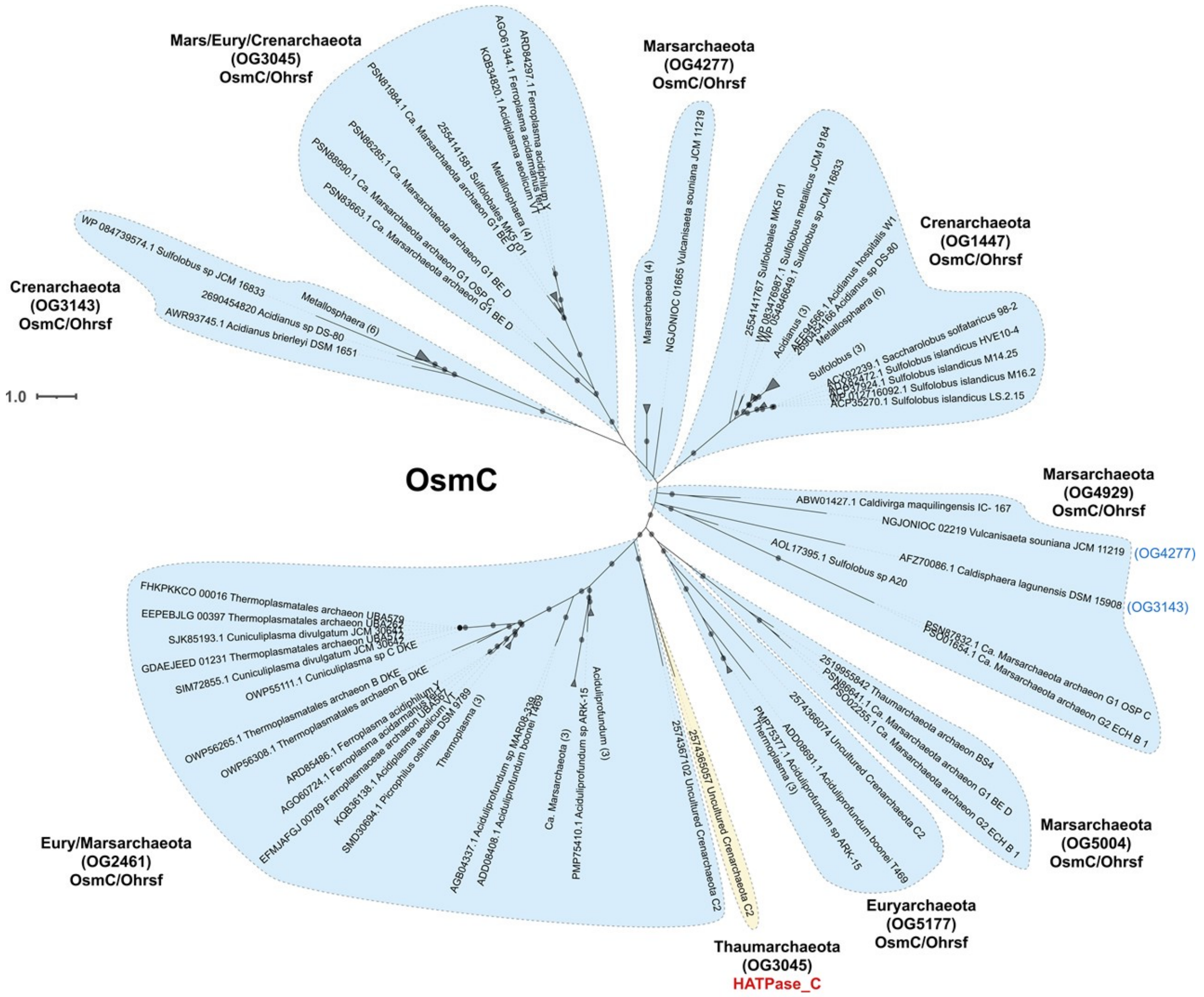

Figure S4 Unrooted phylogenetic tree constructed from the predicted amino acid sequences of OsmC in the different acidophilic Archaea. Each clade is delimited by a blue background. The specific phyla with sequences in each clade are annotated with the corresponding orthogroup. In yellow is shown the sequence of Thaumarchaeota that doesn't share the main functional annotation of OsmC of the other sequences Bootstrap values over 60 are represented with gray circles in the respective branches. Scale bar represent 0.1 amino acid substitutions per site.

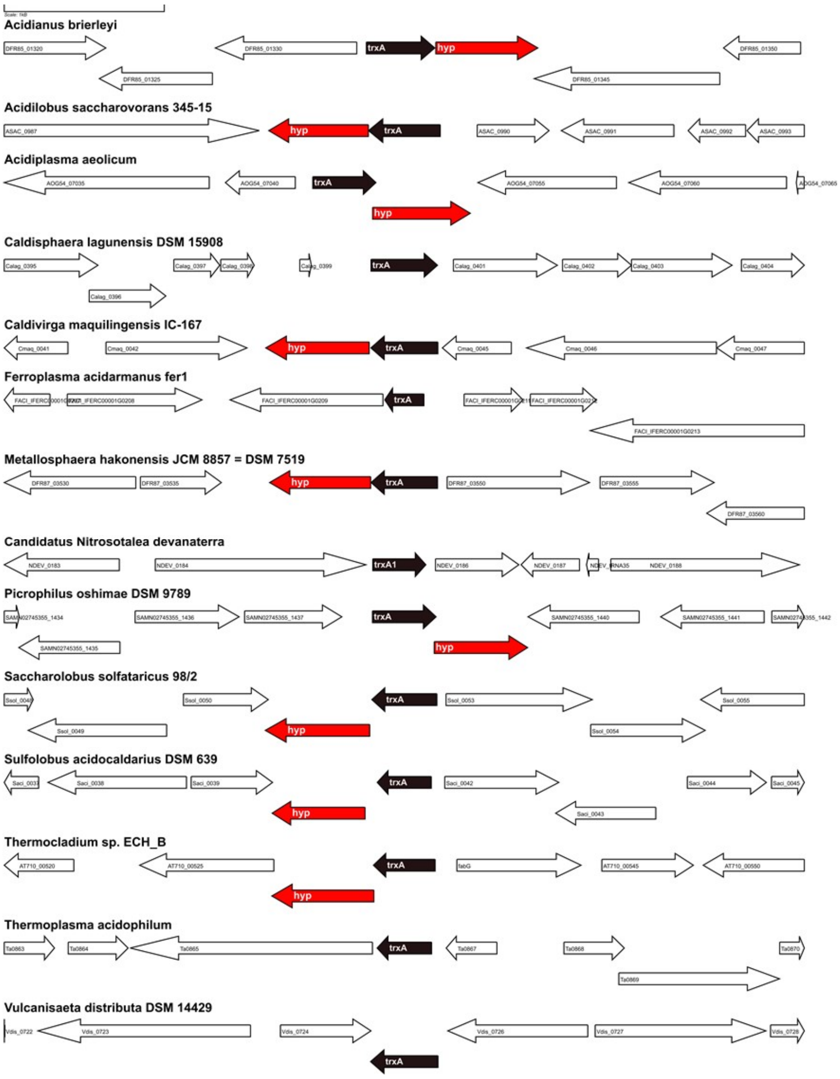

Figure S5. Genomic context of *trxA* and the conserved hypothetical protein OG0923 in several representative genomes. Locus tag for genes are shown inside each of the arrows. Color Coding: red=hypothetical OG0923, black = *trxA*. White: non-conserved genes
